# Strengthening data, analytic and scientific writing skills: Insights from working with 17 health and demographic surveillance system (HDSS) centres in sub-Saharan Africa and South Asia

**DOI:** 10.1186/s12963-026-00495-0

**Published:** 2026-07-28

**Authors:** Tshegofatso Seabi, Beth A Tippett Barr, Chodziwadziwa Whiteson Kabudula, Kobus Herbst, Daniel Ohene-Kwofie, Jean Bashingwa, Audry Dube, Kathleen Kahn, Stephen Tollman

**Affiliations:** 1 South African Medical Research Council, School of Public Health, Faculty of Health Sciences, University of the Witwatersrand Rural Public Health and Health Transitions Research Unit (Agincourt), University of the Witwatersrand, Johannesburg, South Africa; 2 Nyanja Health Research Institute, Salima, Malawi; 3 Africa Health Research Institute (AHRI), uMkhanyakude district, Kwa-Zulu Natal province South Africa

**Keywords:** COVID-19, data management, process paper, scientific writing, SARS‑CoV‑2, Mortality

## Abstract

In the ever-evolving landscape of global health, Health and Demographic Surveillance Systems (HDSS) generate rare and valuable longitudinal data for understanding health and population dynamics and their determinants in low- and middle-income countries, where vital registration systems are often weak. However, the utility of HDSS centres relies heavily on the capabilities of those responsible for cleaning, describing and analysing the wealth of data they generate. This paper describes our targeted strategy for strengthening the capacity of personnel in HDSS research settings, particularly focusing on data managers and analysts. Additionally, we explore the unique challenges created by the COVID-19 pandemic for HDSS research centres, emphasizing the need for a skilled workforce to navigate complex data dynamics. Our approach involved three in-person workshops dedicated to data preparation, analysis, and scientific writing, supplemented by weekly online check-ins and a 25-week writing program. Each workshop included two representatives from participating sites with skills aligned to the workshops. Following two years of intensive capacity building, this project has yielded 15 original research manuscripts currently under review, forming part of a journal special issue on the impact of COVID-19 on mortality in African and South Asian populations. Open-access aggregated data will complement these papers.

**Supplementary Information:**

The online version contains supplementary material available at 10.1186/s12963-026-00495-0.

## Introduction

Health and socio-demographic surveillance systems (HDSS) play a critical role in providing population-based data in environments where vital registration systems do not function effectively [1–3]. These sites have helped document the changing sub-national mortality and cause-of-death profiles that reflect rapid yet complex health transitions [4–6]. They have also been useful in evaluating the impact of policies and programs such as the introduction of new vaccines [1, 11]. By definition, HDSS sites/centres are population-based and reflect the wider population within which they are embedded; the size of the denominator population is largely determined by the scientific questions posed and it is thus not uncommon for denominator populations to be enlarged over time [1, 5]. Mortality data collection and mortality trend analyses are made possible by conducting house-to-house systematic enquiry into death events (coupled with an enquiry into pregnancy outcomes and migration) every three to twelve months [2, 7–9]. This rigorous collection of vital events data in a defined study catchment area is coupled with an enquiry into household membership to ensure population denominators are kept up to date, in order to compute mortality and other epi-demographic rates and trends. In essence, an HDSS is a ‘whole population cohort’ and many are operating in under-resourced settings across sub-Saharan Africa and South Asia. While mostly rural, a growing number of HDSS’s have been established in urban environments, and play a key role in supporting vaccine-based interventions and policy evaluations [10, 11]. Networks such as The International Network for the Demographic Evaluation of Populations and their Health (INDEPTH) [2, 11], Analysing Longitudinal Population-based HIV/AIDS data on Africa (ALPHA) [12], The South African Population Research Infrastructure Network (SAPRIN) [13] and the African Population Cohort Consortium (APCC) have been effective in networking HDSS-based systems and their scientists.

In the context of COVID-19, despite the seemingly high reported death rates globally, it has been argued that reported mortality significantly underestimated the true impact of COVID-19, particularly in low- and middle-income countries [14, 15]. A fundamental question, then, is what was the impact of the COVID-19 pandemic period on mortality, and how much excess mortality occurred in sub-Saharan Africa and South Asia where civil and vital registration systems (CRVS) are weak? HDSS sites with ongoing mortality surveillance and robust population denominators pre- and post-COVID-19 allow us to measure the direct and indirect effects of COVID-19 on mortality in large population cohorts.

HDSS data are robust and context-specific, which has the potential to generate answers to these questions. However, HDSS’s often face the challenge of underutilisation, with a paucity of publications reflecting the wealth of information at their disposal. This can be attributed, in part, to the complex nature of the data they produce, and the insufficient skills of HDSS data managers and analysts to maximize data use for public health action.

As the world strived to fully understand the impact of COVID-19 on health systems and government responses more broadly, it became evident that challenges extend beyond the virus itself. The pandemic underscored the importance of a skilled epidemiological and statistical workforce equipped to both process and understand the implications of data generated during a health crisis. Not only was there a sudden and urgent need for proficient data management in the context of COVID-19, but the indirect effects on mortality and healthcare across diverse settings demanded a nuanced analytical approach that only a well-trained workforce could provide. Data professionals, including data managers and analysts, constitute the backbone of any successful health information system. Their roles extend beyond mere data handling; they are instrumental in translating raw data into actionable insights that inform public health policies and interventions.

In this paper, we present our collaborative, multicentre process to strengthen data management and analytic capacity, and scientific writing skills of key staff in HDSS’s across sub-Saharan Africa and South Asia through a process aimed at elucidating the impact of COVID-19 on mortality in LMICs. By enhancing the skills of those working directly with HDSS data, we can unlock the full potential of the centres, fostering a culture of research and data dissemination that contributes to the broader understanding of population health, and increases the self-sustaining capacity of HDSS’s. We describe the evolving process of capacity-building and strengthening in the project. Through this lens, we contribute to the ongoing discourse on capacity-building and strengthening through HDSSs, positioning data managers and analysts as key agents in the pursuit of comprehensive and accurate data and high-quality publications from sub-Saharan Africa and South Asia. This effort fortified HDSS foundations and helped develop a workforce more capable of adapting to unforeseen challenges, contributing meaningfully to global health knowledge .

## The birth of the “Excess COVID-19 mortality in HDSS’s” project

In a discussion with the Bill and Melinda Gates Foundation (BMGF) in first quarter of 2021, it became evident that an understanding of the impact of the COVID-19 pandemic on all-cause mortality in Africa and South Asia was vitally hampered by the unavailability of data needed for an effective response strategy. Accessing existing longitudinal data pre- and post-COVID-19 would help fill this void and provide an opportunity to strengthen mortality surveillance capacity at HDSS sites. BMGF recognised HDSS sites as an ideal source of accurate mortality count numerator, coupled with robust population denominator data, which could be used to complement other targeted efforts to assess excess deaths during the COVID-19 pandemic, such as burial site surveillance and mobile phone surveys [4, 5]. This initiative aimed to characterise all-cause mortality rates and trends, by age and sex, across a range of rural and urban sub-Saharan African and South Asian settings, all of which had been under continuous health and demographic surveillance since well before the COVID-19 pandemic struck. Specifically, the key analysis objectives were to:

1. Generate empirically derived, comparable mortality trends from 2015 to 2021 across all age-sex groups in rural and urban HDSS settings of sub-Saharan Africa and South Asia, including site-specific and pooled data.
2. Compare the 2015–2019 pre-pandemic mortality rates with the 2020–2021 pandemic mortality rates across age-sex groups in the same rural and urban settings of sub-Saharan Africa and South Asia, to quantify excess mortality during the pandemic.
3. Develop high-quality datasets for centres to make publicly accessible, which can contribute to national, regional and global understanding of the pandemic’s impact.

Once these objectives were developed, a breadth of African and Asian HDSS sites known to the study team to have data of reasonable quality for at least five years preceding the onset of the pandemic were contacted to determine interest in participation. A process of site selection, based on review of the trend data and quality indices was then undertaken.

## Selection of HDSS centres

We planned to include at least 10 HDSS sites across sub-Saharan Africa and South Asia that had ongoing and effectively monitored mortality surveillance from 2015 to 2019 as pre-COVID years, and 2020–2021 as the COVID years of highest mortality, before widespread immunity was acquired. When combined with accurate denominator information on the population under continuous surveillance, the mortality count as numerator makes possible the calculation of accurate mortality rates and trends by sex and age-group.

HDSS centres were selected using six criteria:

1. Geographic location
2. Size of population under surveillance
3. Documented consistent performance in generating mortality data across age-sex groups, with
4. Data that satisfied strict quality assurance measures thereby ensuring internal consistency of site-specific data, along with
5. High data comparability with other HDSS’s, and
6. Stated commitment to contribute anonymized mortality data to a public access data repository

Thirty-two (32) HDSS sites across sub-Saharan Africa, South and Southeast Asia were invited to participate in an initial survey on their activities, particularly mortality surveillance (Questionnaire in Appendix 1). Twenty-six (26) centres responded and completed the initial survey. Additional information gathered included the availability of cause of death data collected through verbal autopsy (VA) (WHO 2016, WHO 2012, site-specific VA tools) and Minimally Invasive Tissue Sampling (MITS). Information from the survey provided investigators with a profile for each of the sites and their host centres, that included population size and geographic location (Additional file 1 &2). Of interest, several of the centres/sites contributed to regional HDSS networks.

Selected criteria were used to identify research centres to participate in the study, and a composite score was computed based on these indicators/metrics (Table 1). A threshold for selecting the sites was established based on the total scores obtained (Table 2). There were 22 sites with a score of *≥* 5.0 which were considered. Following clarification of survey results with sites and discussions between investigators and the BMGF program officers, thirteen (13) centres were determined to be eligible. In addition to the 13 sites, discussion with the Child Health and Mortality Prevention Surveillance (CHAMPS) project resulted in five of their African sites underpinned by an HDSS joining the initiative. Table 3 below lists all sites included in the COVID-19 mortality initiative.^1^

**Table 1:** Mortality indicators and scores for HDSS site selection

Metric	Assigned score
Mortality data collection ongoing?	1.0
Availability of all-cause mortality data	1.0
Availability of all-cause mortality data for all ages	1.0
Period for which mortality data available	Period/Max
Availability of verbal autopsy data for all ages	1.0
Period for which verbal autopsy data available	Period/Max^1^
Willingness to share anonymized data	1.0
Ethical clearance for HDSS	1.0

**Table 2:** Scoring categories for mortality metrics

Score category	Total score
Likely to be selected	8.0
Highly probable	6.9 to 7.9
Less probable	5.0 to 6.8
Unlikely to be selected	< 5

Following the selection process, centres were invited to participate in the initiative. It was expected that they would standardize the data for pooled analysis and contribute additional data to conduct further site-specific analyses. Data preparation specifications and format were sent out to all sites/centres. Data managers at each site were expected to extract data from their site’s operating database into a common standard format (Appendix 1) with assistance from the project technical leads.^2^ The data consisted of demographic, mortality and verbal autopsy information on all individuals from 2014 to the most recent time point (2021). The data contained variables that allowed for analysis of population mortality risk by age, sex, and cause of death. The data preparation process was drawn from the *INDEPTH Network I-Share* data harmonisation and quality assurance process described by Sankoh and Byass (2012) [1]. This included capacity building and strengthening one-to-one sessions with site data managers; and virtual data preparation sessions every two weeks with site-level data managers to extract data into the format specified and as preparation for the first in-person workshop.

## Data preparation & harmonisation workshop – March 2022

Going into this project, we anticipated that data managers and analysts would be well-equipped and experienced both in skills and resources to produce data in the common standardised format required. The original pre-workshop plan was for data specialists or managers from each participating centre to extract individual event histories and verbal autopsy data from their site’s operating databases as described in the data specification and bring the data to the workshop to facilitate standardizing of variables. While the project technical and analytic leads expected some challenges in standardizing the data for a pooled analysis, a marked gap in complex data management and analysis skills soon became evident, notably for large population cohort analyses. This skill deficit was unexpected as we were working with HDSS centres that had collected longitudinal population data for years and some for decades. In our assessment, any capacity-building initiative would need to be tailored to the specific needs of HDSS data managers and analysts, encompassing advanced data management techniques, statistical analysis, and a deep understanding of the unique challenges posed by health crises such as the ongoing COVID-19 pandemic.

The newly identified gap necessitated a data preparation workshop, preceded by biweekly online group sessions with data managers to troubleshoot problems and facilitate collaborative learning. Realising the extent of capacity needs across the centres, online sessions were held with individual sites to review data specifications and provide tailored site-specific support. Simultaneously, data quality checks were carried out on all data submitted and data managers were in ongoing communication to confirm that datasets after quality checks reflected valid site-specific information. This process of data extraction and quality checking began in late January 2022 and continued through an in-person workshop in March 2022.

Dr. Chodziwadziwa Kabudula of the SAMRC/Wits Rural Public Health and Health Transitions Research Unit (Agincourt) and Dr. Kobus Herbst of the South African Population Research Infrastructure Network (SAPRIN) co-hosted a week-long, in-person workshop with 30 participants from the HDSS centres. Initially conceived as a data harmonisation workshop, it was revised to accommodate basic data preparation and skills-building for data managers. The workshop was held from 28 March-1 April 2022 at the University of the Witwatersrand in Johannesburg, South Africa (Additional file 3). The purpose was to strengthen each data manager’s capacity to extract data into a common standard format. This meant familiarising the research teams with the data collection system and quality assurance standards (which became an iterative process throughout the workshop), harmonising the data to enable comparisons across sites, and reviewing both individual and pooled sites’ historical mortality data. Both the lead team and data managers across the multicentre platform learned much about the data which extended to sites’ current data quality relative to the quality required for harmonisation.

Initially, data managers expected to hand over their site data for others to clean and analyse while receiving lecture-style sessions on data management (their experience in previous workshops hosted by coordinating networks). This approach may well have contributed to HDSS data scientists remaining unaware of their own capacity gaps in data management and analysis. Thus, the leadership team instituted a hands-on capacity-building approach to data preparation. Workshop activities included presentations on data collection and analysis methods, followed by breakaway sessions for participants to organise their data, compare, and assist other teams, creating a conducive space for learning, support, and collaboration among the data managers (Additional file 3).

## Data cleaning & data management

Following the workshop, there were bi-weekly progress check-ins online with the group, and site-specific meetings for those needing specialised support. Some centres were visited in person by Dr Kabudula to solve data challenges and accelerate capacity-building in data management. The workshop and biweekly sessions proved valuable to data teams, ensuring dedicated time to focus on data quality while learning different approaches to data management through networked collaborations.

Anticipating a transition from data management to analysis, HDSS data analysts and scientists joined the fortnightly calls. Initially, preliminary pooled and individual site results were presented during the fortnightly sessions for all to review and discuss – which helped check the consistency of findings with the contextual understanding of site data analysts. This became an iterative process of quality checks, initial analyses, presentation and interrogation to ensure that the harmonized data being used accurately represented each setting.

Preliminary findings were presented to an International Advisory Group in mid-2022. The Advisory Group^3^ contributed valuable feedback on the data and analytic approaches being applied. It became clear that COVID-19’s impact on mortality varied markedly by setting, hence the importance of site/centre-specific analyses to better understand who was affected and possible reasons why.

## Data analysis workshop – december 2022

Picking up from the online biweekly check-ins and advisory board meetings, the workshop (with at least a data manager and analyst from each site) focused directly on analytic approaches. The primary purpose of this workshop was a thorough analysis of HDSS platform data to properly understand the extent (or not) of excess mortality across contexts. In addition, to begin comparative efforts to better understand regional patterns of mortality across the geographies represented Workshop activities included presentations on data collection and analytic methods, coupled with breakaway sessions for site-specific and multisite collaborations (Additional file 4). This approach helped establish an environment conducive to learning, mutual support, and collaborative efforts among data managers, analysts, and scientists.

## Capacity-building for site-specific analyses and scientific writing

In December 2022, a lecture titled “The A-to-Z of Scientific Writing” was given by Dr Beth Tippett Barr of Nyanja Health Research Institute in Malawi, with the understanding that centres would utilize their now clean data and analytic skills to create their site-specific papers. A pre-workshop assessment revealed great enthusiasm, albeit over half of the participants had not previously first-authored a peer-reviewed article, and several had never co-authored a manuscript.

Recognising that skills-building in scientific writing would be required, a 16-week online short course was devised to guide the participants through the entire process of drafting a manuscript, from conceptualization to publication (Additional file 5). To help first authors protect their learning and writing time during working hours, the recommendation was to block four hours every week for an online conference call. Each session would begin with a lecture and discussion, the remaining being used to apply what was learned and make consistent progress on manuscript preparation.

### Rapid assessment

To prepare, participants completed an online survey that included an in-depth assessment of past experience, which steps in manuscript development would be helpful, which times were suitable for the weekly calls, what additional data sources could be drawn on for further analysis, and their idea for a manuscript including an outline of their research question and analysis plan. The initial assessment included 17 HDSS centres from 9 countries in sub-Saharan Africa and South Asia, spanning six time zones. When the course started, 14 centres had committed to participating.

From this information, it was clear that support would be required for developing a statistical analysis plan and conducting analyses, in addition to learning about the writing process. As a result, the original 16-week writing course was expanded to 25 weeks (Additional file 6), the idea being that by the end, every site would have a draft manuscript, with coauthor and institutional approvals secured.

### The writing course process

Before the writing course, the technical and writing leads worked with participants to understand the scope of their data resources to provide additional insight into COVID-19 mortality in their catchment areas.

During the early weeks of the course, we provided didactic lectures on how to review scientific and grey literature, use reference management software, and develop an analysis plan. This helped participants understand how their analyses could fit into the global picture, Concurrently, data merging and cleaning were finalized with the analytic teams to prepare for site-specific analyses.

After some months, it became clear that further flexibility in course content and time was needed. As noted, over half the participants were new to first authorship, almost all had English as a second language, and the time needed for iterations of drafts varied widely between participants. As these papers were contributing to a journal supplement, the challenge became keeping those with fewer skills aligned with those further ahead.

### Mid-course writing retreat

In August 2023, the approximate midpoint of the course, we held an in-person writing retreat at the University of the Witwatersrand in Johannesburg (Additional file 5). With didactic sessions already conducted remotely, the writing retreat time was used to bring the site writing leads and data analysts together to finalize a first draft. The facilitators conducted mid-week reviews of every manuscript and provided guidance for completion by the end of the week-long retreat. This proved highly successful with almost all centres ready for a first review by coauthors at the close of the workshop.

The second half of the writing course aimed primarily to protect writing time for participants and ensure availability of facilitators and mentors to provide rapid responses to questions and concerns. Using Microsoft Teams for web-based conference calls meant that multiple side meetings could be conducted in parallel. In a couple of months, 12 of 14 manuscripts were completed and approved by co-authors, with institutional approval pending for about half and subsequently granted. In November, we held a final didactic session on formatting in Microsoft Word and Excel and worked with the site authors to format their manuscripts to suit Population Health Metrics journal requirements.

### Engaging and maintaining support of HDSS site leadership

The engagement of HDSS centre leadership, usually the PI, was a strategic component of the plan. We needed their buy-in for the half-day each week that participants would spend in our virtual sessions, that they would be responsive to our timelines during the iterative review process, and willing to assist in case of any delays in the coauthor and institutional review processes. We held three calls with site leadership throughout the course and shared with them a “Book of Abstracts” that was compiled after the midpoint writing retreat. This proactive engagement with site leadership successfully facilitated site-level progress, ensuring the manuscripts were all completed by the end of November.

### Challenges and gaps in site capacity

We found three key gaps in site-level capacity during this process: Data management, including data cleaning and the standardization of data for pooling; data analysis; and scientific writing skills.

### Data management capacity

Although there are variables in common, a key issue is currently; there are no standardised approaches to data collection across HDSS centres in sub-Saharan Africa and South Asia [13]. Each site may adopt unique methodologies, leading to differences in data collection processes, and considerable effort is needed to harmonise data. These variations encompass differences in the definition and categorisation of variables, posing challenges for data managers and analysts when harmonising and aggregating data for broader, cross-site analyses. For this project, most HDSS centres typically employed longitudinal study designs, tracking changes within populations over time. However, a challenge arose when distinguishing between longitudinal and repeated cross-sectional data within and across some sites. Inconsistent application of this basic database design principle created notable challenges in standardising data. Data managers and analysts faced the task of navigating these distinctions, impacting the accuracy and reliability of temporal analyses and hindering the ability to draw meaningful (and comparable) conclusions from the data.

Another central challenge involved variation in how key variables are defined and measured across HDSS sites. Discrepancies in definitions – for example, age groups or socioeconomic status (SES) – impede cross-site comparisons and limit the generalizability of research findings. Data managers and analysts had to grapple with the complexity of reconciling these variable differences, which required meticulous attention to detail and advanced statistical techniques to ensure the validity and reliability of analytical outcomes. Hence, time and effort were needed to ensure data in this project was standardised and harmonised to enable accurate and comparable analytic outcomes.

#### Data cleaning and analysis

A related challenge was the capacity required for thorough data cleaning. Selection of sites considered the availability of data but not an assessment of the capacity of sites to clean or analyse the data. In executing the activities of the project, we learned that most centres had limited resources, both human and technological. Hence, great effort was devoted towards building capacity for meticulous identification and rectification of errors, outliers, and inconsistencies in the datasets. This also speaks to strengthening capacity in the analysis of longitudinal datasets and writing of peer-reviewed articles.

#### Scientific writing

In the initial assessment, several participants expressed concern about their language skills, especially for scientific writing, as almost all participants learned English as a second, third or fourth language. We found however that English language skills were above average, and instead what was needed was learning the specifics of scientific writing including: How to organize an introduction to lay out a logical flow and argument; how to describe methods concisely yet with enough detail to replicate the study; how to describe tables and figures of results without repeating the data verbatim; and how to draft a discussion that succinctly expressed the importance of their findings, and connected back to the introduction and objectives of the study. These are challenges faced by new first authors regardless of background and education. Given the eagerness of participants to learn how to write academic papers, and their commitment to the weekly blocked writing time, it was relatively straightforward to successfully walk them through the process over some months.

## Lessons learned

Among the key lessons was the critical need for intentional and targeted skills-building initiatives for data analysis and scientific writing across all HDSS centres – as the proficiency of data managers and analysts directly influences the quality of research outcomes. From experience, we are confident the data managers and analysts valued the time dedicated to training and improvement.

Restrictions imposed by COVID-19 and forced adaptation to remote working have provided a platform in which online work is normal. While this project involved both in-person and virtual sessions, the online sessions occurring between in-person workshops have proven highly effective in enhancing data analysis and scientific writing skills. This lesson acknowledges the adaptability of teams and the strengths of carefully structured online sessions, enabling participants from diverse locations to engage in collaborative learning. This not only addressed geographical constraints but also fostered a sense of inclusivity and collaboration among data professionals and scientists across HDSS centres.

We found that the weekly dedicated time in the virtual sessions was important both for the analysis and writing aspects of the project. Equally, this fostered a culture of accountability and shared momentum amongst the group, which was reinforced by robust support from writing and technical teams. Sustained commitment and endorsement at the highest levels of HDSS leadership are integral to the success of data analysis and publication endeavours.

Flexibility in the analysis and writing approach that allows for an iterative process should be considered when setting timelines. A mechanism to track progress and assist in goal setting and accountability is important. This was born out of the experience: allocating additional time is vital to accommodate the iterative review and refinement process with co-authors. Moreover, this allows for thorough centre clearance procedures, ensuring that research outputs meet the standards of accuracy, consistency, and scientific rigour before dissemination.

Altogether, these lessons from insights can serve as guideposts for fostering a culture of innovation and research excellence within the realm of health and demographic surveillance platforms across sub-Saharan Africa, South Asia and beyond.

## Conclusion

The experience gained from the process of standardizing, harmonizing and pooling the HDSS data collected in sub-Saharan Africa and South Asia underscores the importance of ongoing learning and adaptation. Lessons learned from data management and analytic efforts provide valuable insights for refining processes, improving methodologies, and enhancing overall efficiency. By intentionally strengthening the analytic and scientific writing skills at HDSS centres, we have contributed to enduring skills that will serve future research as well as the quality of the data outputs of this project. This is essential to sustain their involvement in the evolving global health conversation.

## Supplementary Information

Below is the link to the electronic supplementary material.

Supplementary Material 1

Supplementary Material 2

Supplementary Material 3

Supplementary Material 4

Supplementary Material 5

Supplementary Material 6

Supplementary Material 7

## Data Availability

Not applicable.

## Footnotes

1 Max = 7 years of data (2015–20210.

2 Drs Kobus Herbst and Chodziwadziwa Kabudula.

3 Advisory Group: Debbie Bradshaw, Samantha Dolan, Sam Clark, Georges Renier.

## Abbreviations

HDSS: Health and socio-demographic surveillance systems
INDEPTH: International Network for the Demographic Evaluation of Populations and their Health
ALPHA: Analysing Longitudinal Population-based HIV/AIDS data on Africa
SAPRIN: The South African Population Research Infrastructure Network
APCC: African Population Cohort Consortium
CRVS: Civil and Vital Registration Systems
HIV: Human Immunodeficiency Virus
AIDS: Acquired Immune Deficiency Syndrome
BMGF: Bill and Melinda Gates Foundation (now Gates Foundation)
WHO: World Health Organization
VA: Verbal Autopsy
MITS: Minimally Invasive Tissue Sampling
LMICs: Low- and Middle-Income Countries
SES: Socioeconomic status
