# Supplementary material for: Strengthening data, analytic and scientific writing skills: Insights from working with 17 health and demographic surveillance system (HDSS) centres in sub-Saharan Africa and South Asia
Source: Popul Health Metr. 2026 Jul 28;23(Suppl 2):80. doi: 10.1186/s12963-026-00495-0 (PMC13420847; doi:10.1186/s12963-026-00495-0)

**Supplemental Figure 1: Showing the population size of sites participating in the initial survey**


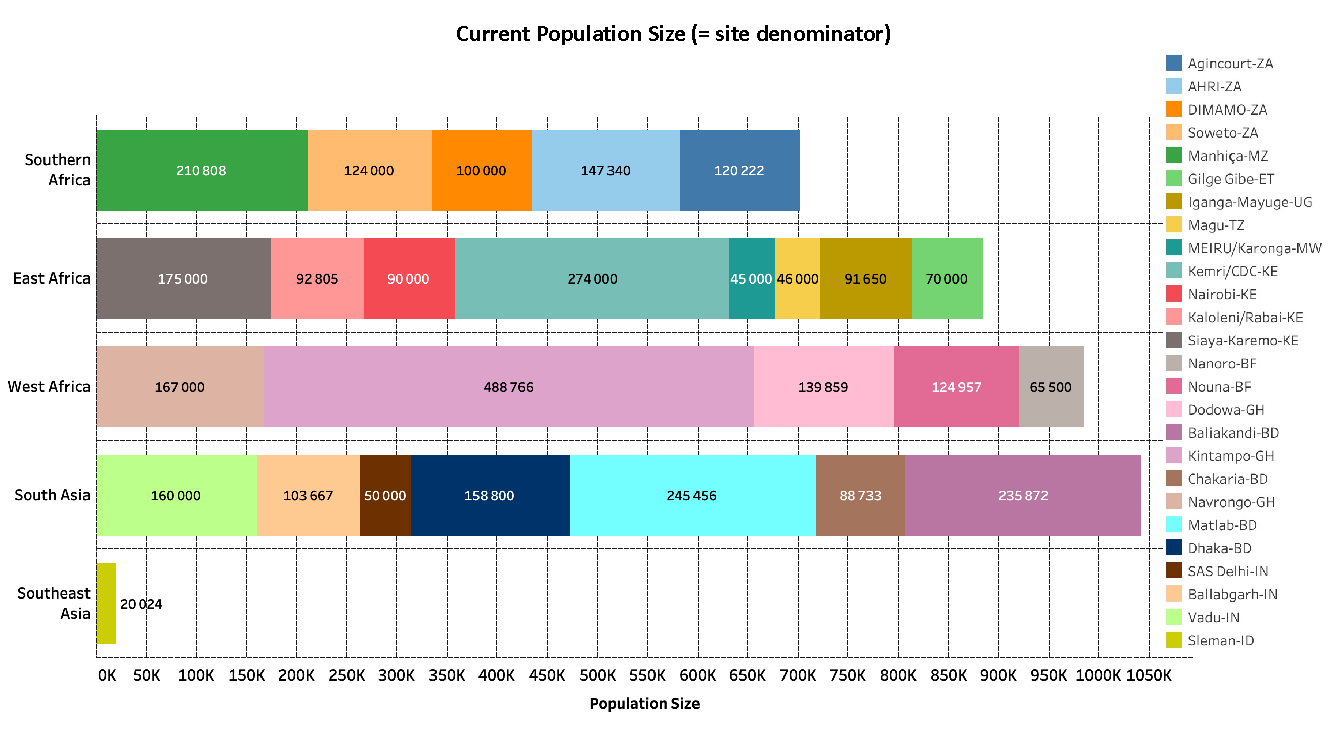

Supplement: Supplementary file 1 — Supplementary Material 1 [file 12963_2026_495_MOESM1_ESM.docx]
