# Supplementary material for: Strengthening data, analytic and scientific writing skills: Insights from working with 17 health and demographic surveillance system (HDSS) centres in sub-Saharan Africa and South Asia
Source: Popul Health Metr. 2026 Jul 28;23(Suppl 2):80. doi: 10.1186/s12963-026-00495-0 (PMC13420847; doi:10.1186/s12963-026-00495-0)

Supplemental Figure 2: Showing the geospatial overview of self-defined settlement types of the participating sites


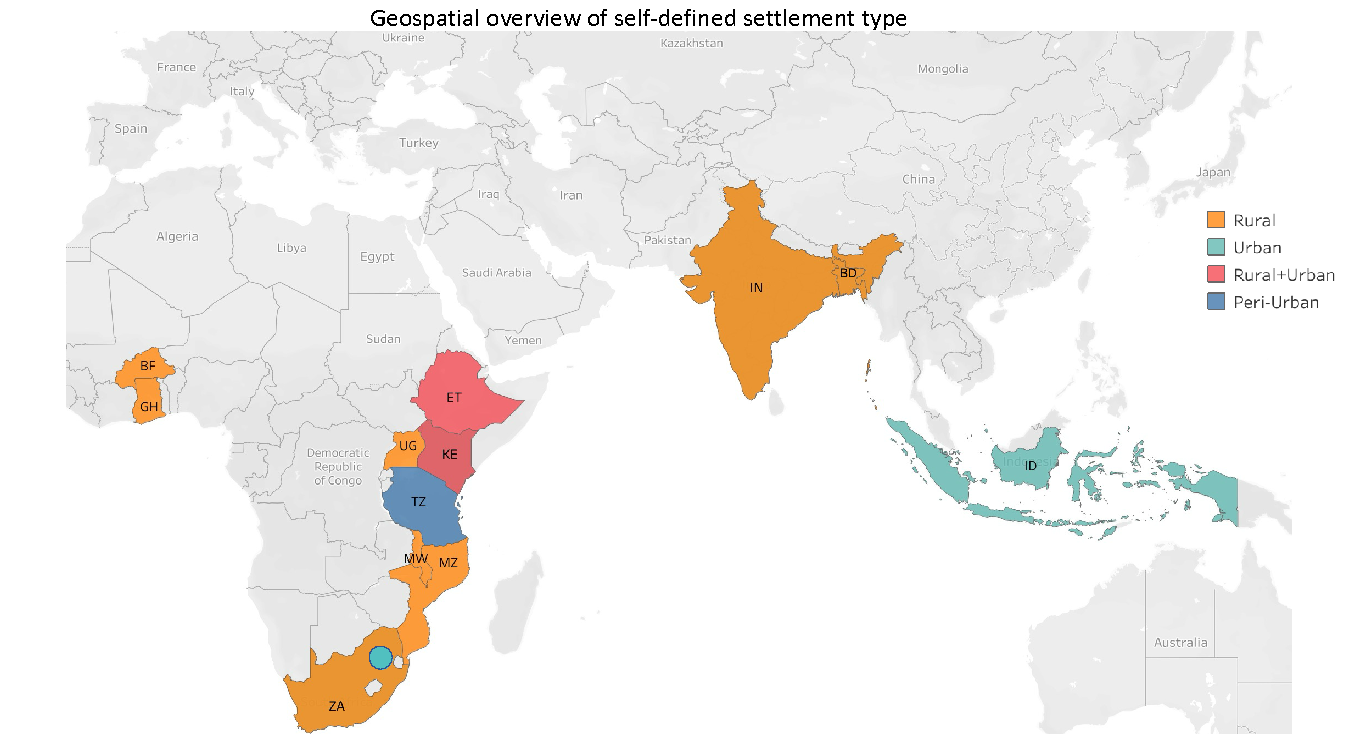

Supplement: Supplementary file 2 — Supplementary Material 2 [file 12963_2026_495_MOESM2_ESM.docx]
