# Supplementary material for: Strengthening data, analytic and scientific writing skills: Insights from working with 17 health and demographic surveillance system (HDSS) centres in sub-Saharan Africa and South Asia
Source: Popul Health Metr. 2026 Jul 28;23(Suppl 2):80. doi: 10.1186/s12963-026-00495-0 (PMC13420847; doi:10.1186/s12963-026-00495-0)
Supplement: Supplementary file 3 — Supplementary Material 3 [file 12963_2026_495_MOESM3_ESM.docx]

**Supplemental Table 1: Data preparation workshop**

**Monday, 28 March 2022**

| 08:30 | Registration and settling into the workshop space | In-person  Resource Centre |
| --- | --- | --- |
| 09:30 | Welcome | Steve Tollman/Tobias Chirwa |
| 09:45 | Housekeeping matters | Tshego Seabi &  Kurium Govender |
| 10:00 | Introduction to the Excess Mortality study | Steve Tollman |
| 10:30 | Introduction to the data preparation workshop | Cho Kabudula |
| 11:00 | **TEA/COFFEE** |  |
| 11:15 | Status of Individuals and Individual Events staging datasets | Daniel Ohene-Kwofie |
| 12:30 | **Quality assurance of staging data sets (1)** | Kobus Herbst |
| 13:00 | **LUNCH** |  |
| 14:00 | **Quality assurance of staging data sets (2)** | Kobus Herbst |
|  | Systematic feedback of quality assurance of Individuals and Individual Events staging datasets |  |
| 17:30 | Return to hotel |  |

**Tuesday, 29 March 2022**

| 09:00 –  17:00 | **Breakout sessions** for sites to resolve data quality issues identified in the Individuals and Individual Events staging datasets |  |
| --- | --- | --- |

**Wednesday, 30 March 2022**

| 09:00 | Introduction to Verbal Autopsy | Kathy Kahn |
| --- | --- | --- |
| 10:00 | Introduction to InSilicoVA | Cho Kabudula |
| 11:00 | **TEA/COFFEE** |  |
| 11:30 | Status of VA datasets | Daniel Ohene-Kwofie |
| 12:30 | **Quality assurance of Verbal Autopsy data (1)** | Cho Kabudula |
|  | Introduction to QA of Verbal Autopsy data |  |
| **13:00** | **LUNCH** |  |
| 14:00 | **Quality assurance of Verbal Autopsy data (2)** | Cho Kabudula |
|  | Systematic feedback of quality assurance of VA datasets |  |
| 18:30-21:00 | Group Dinner |  |

**Thursday, 31 March 2022**

| 09:00 | Breakout sessions for sites to resolve data quality issues identified in the Verbal Autopsy datasets |  |
| --- | --- | --- |

**Friday, 01 April 2022**

| 09:00 | **Wrap-up session on data preparation** | Kobus et al. |
| --- | --- | --- |
|  | Reviews of harmonized data |  |
|  | Next steps |  |
| 13:00 | Lunch and closing |  |
