# Supplementary material for: Strengthening data, analytic and scientific writing skills: Insights from working with 17 health and demographic surveillance system (HDSS) centres in sub-Saharan Africa and South Asia
Source: Popul Health Metr. 2026 Jul 28;23(Suppl 2):80. doi: 10.1186/s12963-026-00495-0 (PMC13420847; doi:10.1186/s12963-026-00495-0)
Supplement: Supplementary file 4 — Supplementary Material 4 [file 12963_2026_495_MOESM4_ESM.docx]

**Supplemental Table 2:** Data Analysis workshop program

Monday, 28 November 2022

| 08:30 | Registration and settling into the workshop space | In-person  Resource Centre |
| --- | --- | --- |
| 09:30 | Welcome | Tobias Chirwa Steve Tollman  Kathy Kahn |
| 09:55 | Housekeeping matters | Tshego Seabi |
| 10:00 | Overview of the Excess Mortality study | Steve Tollman |
| 10:30 | Introduction to the data analysis workshop | Cho Kabudula |
| 11:00 | **TEA/COFFEE** |  |
| 11:15 | Status of **Individuals** and **IndividualEvents** staging datasets | Kobus Herbst |
| 12:30 | **LUNCH** |  |
| 13:30 | Breakout sessions for sites to resolve data quality issues identified in the **Individuals** and **IndividualEvents** staging datasets | Kobus Herbst Cho Kabudula  Jean Bashingwa |
| 15:00 | Introduction to writing  Planning the outputs | Beth Tippett-Barr |
| 17:30 | Return to hotel |  |

**Tuesday, 29 November 2022**

| 09:00-12:30 | Demonstration of methods for computing overall and age specific mortality rates, life expectancy and decomposition of life  expectancy by age and sex. | Cho Kabudula |
| --- | --- | --- |
| 12:30-13:30 | Lunch |  |
| 13:30-17:00 | Breakout sessions for participants to apply methods on their site- specific data | Jean Bashingwa  Cho Kabudula |

**Wednesday, 30 November 2022**

| 09:00-12:30 | Demonstration of methods for estimating excess mortality using time  series technique. | Jean Bashingwa |
| --- | --- | --- |
| 12:30-13:30 | Lunch |  |
| 13:30-17:00 | Breakout sessions for participants to apply methods on their site-  specific data | Jean Bashingwa  Cho Kabudula |
| 18:30-21:00 | Group Dinner |  |

**Thursday, 01 December 2022**

| 09:00 | Advanced presentation on writing | Beth Tippett-Barr |
| --- | --- | --- |
| 12:30-13:30 | LUNCH |  |
| 13:30-16:00 | Writing session and preparation of site-specific presentations | Cho Kabudula Kobus Herbst Jean Bashingwa  Beth Tippett-Barr |
| 15:00 | TEA/COFFE |  |
| 15:30 | Presentation of site-specific results | Site 1-6 |

**Friday, 02 December 2022**

| 08:00 | **Wrap-up session on data preparation** | Kobus et al. |
| --- | --- | --- |
|  | Continuation of presentations on site-specific results | site 7-18 |
|  | Next steps – plans for publication and ongoing support |  |
| 12:45 | Closing remarks | Solveig Argeseanu  Steve Tollman |
|  | LUNCH |  |
