# Supplementary material for: Strengthening data, analytic and scientific writing skills: Insights from working with 17 health and demographic surveillance system (HDSS) centres in sub-Saharan Africa and South Asia
Source: Popul Health Metr. 2026 Jul 28;23(Suppl 2):80. doi: 10.1186/s12963-026-00495-0 (PMC13420847; doi:10.1186/s12963-026-00495-0)
Supplement: Supplementary file 5 — Supplementary Material 5 [file 12963_2026_495_MOESM5_ESM.docx]

**Supplemental Table 4:** Writing workshop agenda

|  | **Monday** | **Tuesday** | **Wednesday** | **Thursday** | **Friday** | **Saturday** |
| --- | --- | --- | --- | --- | --- | --- |
| **8:30-10:30** | Introductions & paper updates (each country presents) | Lecture: Formatting papers in Word and tables in Excel | Work session: Writing time | Work session: Writing time | Lecture: Data dissemination & scientific presentations | Work session: Writing time |
| **10:30-11:00** | Tea Break | | | | | |
| **11:00-1:00** | Lecture: How to use Zotero referencing software | Work session: Format papers using journal guidance | Work session: Writing time | Lecture: Finding a conference and writing an abstract | Work session: Writing time | Work session: Writing time |
| **1:00-2:00** | Lunch | | | | | |
| **2:00-5:00** | Work session: Application of referencing software to participant papers* | Work session: Complete referencing and formatting | Work session: Writing time | Work session:  Write abstract for target conference | Work session: Writing time | Status updates  Q&A |
|  |  |  |  |  |  |  |
| **During working sessions, participants can request side meetings with facilitators* | | | | |  |  |
