# Supplementary material for: Strengthening data, analytic and scientific writing skills: Insights from working with 17 health and demographic surveillance system (HDSS) centres in sub-Saharan Africa and South Asia
Source: Popul Health Metr. 2026 Jul 28;23(Suppl 2):80. doi: 10.1186/s12963-026-00495-0 (PMC13420847; doi:10.1186/s12963-026-00495-0)
Supplement: Supplementary file 6 — Supplementary Material 6 [file 12963_2026_495_MOESM6_ESM.docx]

**Supplemental Table 3:** 25-week writing course plan and expectations

| **25-week writing course & workshop** | | | | | | |
| --- | --- | --- | --- | --- | --- | --- |
| **Wk** | **Hrs** | **Lecture** | **In-session work** | **Participant assignment due before session** | **Mentor assignment due before session** | ***Weekly objectives & Flow*** |
| 1 | 4 | Literature review | Literature review | N/A | N/A | *Know the literature before developing an analysis plan - understand how your idea fits into what is/isn't known about the topic. Identify any specific knowledge gaps you may be able to fill with your analysis.* |
| 2 | 4 | Analysis plan | Analysis Plan | Draft manuscript no matter how short or incomplete | N/A | *Plan your analysis before doing it - begin with a plan and follow it through - don't get distracted by interesting things that pop up during your analysis. Make a note to follow those up later with a subsequent analysis/paper.* |
| 3 | 4 | No lecture.  1:1 consults available in "breakout rooms".  Full participation expected in online working time. | Data analysis | Analysis plan with shell tables shared with course facilitators and Sr. Author/PI for review |  | *Start work on the analysis. Statistician(s) available for side meetings. Expect to do additional analysis work before next week* |
| 4 | 4 | No lecture.  1:1 consults available in "breakout rooms".  Full participation expected in online working time. | Data analysis | As soon as completed, share completed tables & figures with Course facilitators, and 2nd and Sr Authors. | Analysis plan reviews & feedback | *Keep working on analysis during and out of class* |
| 5 | 4 | No lecture. Quick updates from participants.  1:1 consults available in "breakout rooms".  Full participation expected in online working time. | Data analysis | As soon as completed, share completed tables & figures with Course facilitators, and 2nd and Sr Authors. | N/A | *Keep working on analysis during and out of class* |
| 6 | 4 | Interpreting your results | Review tables/figures. List of key findings (or important lack of findings) & relevance. | Table shells - share for review and feedback | Review table shells and completed tables/figures as they're submitted, through to the end of June | *Critical thinking about the analysis results. Make that connection between your results and the global literature.* |
| 7 | 4 | Writing up your results | Revise table shells | N/A | N/A | *Share results and thoughts about results. Get feedback from peers and mentors* |
| 8 | 4 | No lecture.  1:1 consults available in "breakout rooms".  Full participation expected in online working time. | Analysis | Table shells - submit for review no later than June 12th | Feedback on data analysis presentations from 1st session | *Share results and thoughts about results. Get feedback from peers and mentors* |
| 9 | 4 | No lecture.  1:1 consults available in "breakout rooms".  Full participation expected in online working time. | Analysis | N/A | Feedback on data analysis presentations from 2nd session | *Learn how to write up your results; positioning your results narrative so that you can add your perspective/opinion in the discussion section* |
| 10 | 4 | No lecture.  1:1 consults available in "breakout rooms".  Full participation expected in online working time. | Analysis | Send completed analysis to Wits team no later than June 30th in order to participate in upcoming workshop | N/A | *Finding that flow from data collection through data analysis - establishing credibility for your results and discussion* |
| 11 | 4 | Data analysis presentations by 1st half of the participants | Presentations | Methods section | Feedback on results sections | *Working time to pull together everything learned so far and complete analysis in advance of the workshop* |
| 12 | 4 | Data analysis presentations by 2nd half of the participants | Presentations | N/A | Feedback on methods sections |  |
| 13 | 4 | Writing your methods section | Methods / Analysis / Results | N/A | N/A |  |
| 14 | 50 | **In-person Writing Workshop.**  ***Objective: Complete 1st draft of manuscript and stakeholder presentation by end of workshop*** | | Draft 1 shared with 2nd and Sr authors by end of workshop - 2-week turnaround time | Iterative review as sections are completed during the week | *Write first complete draft* |
| 15 | 0 | No meeting | N/A | Share abstract with Beth for inclusion in 'Workshop abstract book' | Compile abstracts for sharing with sites | *Learn that 'red ink' is a good thing; multiple iterations are a necessary evil to get your paper to the publication stage.*  *Mentors - guide your mentee through this stage; ensure they don't lose momentum or focus. They may need help with follow-up up co-author feedback, especially if its a senior colleague not responding to them.* |
| 16 | 0 | No meeting | N/A | Remind co-authors of feedback due date | Call with Site PI's to emphasize need for timely feedback |  |
| 17 | 4 | Work on coauthor comments, Q&A | Revisions to draft | Draft 2 shared with all co-authors for review w/in 10 days | No review of draft 2 (wait for 3rd version).  Provide feedback on abstracts by end of week |  |
| 18 | 4 | Work on revisions, Q&A | Revisions to draft | 1. Email follow-up with co-authors to secure feedback  2. Review edited abstracts & submit changes by start of session | Check in to ensure follow-up with co-authors is done.  Call with Site PI's to update them on progress and request support to maintain momentum. |  |
| 19 | 4 | Participant updates, Q&A | Revisions to draft | Submit Draft 3 to 2nd/Sr authors, **and submit for institutional clearance** | Check in to ensure papers were submitted for clearance |  |
| 20 | 4 | Participant updates, Q&A | Revisions to draft |  |  |  |
| 21 | 4 | Participant updates, Q&A | Revisions to draft | Email follow-up with co-authors | Review draft 3 - actively help with "polishing" | *Final stages ensuring everyone has completed revisions, has institutional approval, and has formatted documents correctly.* |
| 22 | 4 | Participant updates, Q&A | Revisions to draft | Revisions |  |  |
| 23 | 4 | Participant updates, Q&A | Revisions to draft | Confirm institutional clearance is in process |  |  |
| 24 | 4 | Group formatting session for Population Health Metrics | Formatting and final touches | Revisions | Actively assist 1st author in following-up institutional clearance. |  |
| 25 | 4 | Group meeting; final Q&A | Formatting and final touches | Revisions |  |  |
|  | 138 |  |  | Journal submission | *~140 hours of group sessions & group work concluded* | |
