# Supplementary material for: Strengthening data, analytic and scientific writing skills: Insights from working with 17 health and demographic surveillance system (HDSS) centres in sub-Saharan Africa and South Asia
Source: Popul Health Metr. 2026 Jul 28;23(Suppl 2):80. doi: 10.1186/s12963-026-00495-0 (PMC13420847; doi:10.1186/s12963-026-00495-0)
Supplement: Supplementary file 7 — Supplementary Material 7 [file 12963_2026_495_MOESM7_ESM.docx]

**Appendix 1:** HDSS-based Mortality Surveillance in Africa and South Asia - Data Specification

**Overall project goal:** Funded by *the Bill & Melinda Gates Foundation,* the goal of this initiative is to characterize all-cause (and cause-specific) mortality rates and trends, by age and sex, across a range of rural and urban sub-Saharan African and South and Southeast Asian settings under continuous health and demographic surveillance in order to understand the excess mortality of the COVID-19 pandemic in key low and middle-income country (LMIC) settings.

**Approach:** In this project, the investigators plan to leverage a carefully selected group of up to 10 well-functioning population-based sites/centers and networks (HDSS or comparable) across sub-Saharan Africa and South and Southeast Asia to document the total numbers of deaths each year from 2015-2019 as pre-COVID years, 2020 as a key COVID year-1 (retrospective), and 2021 as a second pandemic year (prospective). This ‘numerator’ information, when combined with accurate ‘denominator’ information on the population under continuous surveillance, makes possible the calculation of mortality rates and trends across age-sex groups.

**Data preparation**

There will be ongoing interaction between study technical leads and lead data specialists at site level – with capacity strengthening ongoing and integral in preparing data for the project.

1. **Approach**

- Data Managers at each site will extract data from their site’s operating databases into a common standard format with assistance from the project technical leads. The data will consist of demographic, mortality and verbal autopsy information on all individuals from 2014 up to the most recent period (2021).
- The data will contain variables that would allow analysis of mortality risk by age, sex, and cause of death.
- The data preparation process will follow the INDEPTH Network I-Share data extraction and quality assurance process described in “Sankoh O, Byass P. The INDEPTH Network: filling vital gaps in global epidemiology. *International Journal of Epidemiology*. 2012 ;41(3):579-88”.

Note: sites are also required to provide COVID-19 related critical contextual information – timelines of waves/peaks, restrictions on gatherings, closure of public transport, stay at home requirements, international travel controls, cumulative proportion of the population vaccinated) (see for example <https://github.com/OxCGRT/covid-policy-scratchpad/tree/master/regional_reports>)

1. **Individual Event Histories**.

The surveillance data associated with a particular individual over the course of his/her exposure to demographic surveillance over the period 2014 – 2021 will need to be extracted into a series of event records as specified in Tables 1 and 2. The first event for any individual will either be enumeration, birth or in-migration followed by a variable number of event records for each observed event associated with that individual, such as out-migration or death.

***Table 1: Individuals***

| **Variable name** | **Description** | **Coding** | **Notes** |
| --- | --- | --- | --- |
| HDSSName | Name of HDSS site | site specific | Character |
| IndividualId | A number uniquely identifying all the records belonging to a specific individual in the data file. | site specific | For data anonymization purposes, this number should not be the same as the identifier used by a contributing centre to identify the individual, but the contributing site should retain a mapping from this identifier to their identifier |
| MotherId | IndividualId of the mother of the specific individual |  |  |
| Sex | Male or female | 1 Male  2 Female | Must not vary between residence episodes |
| DoB | The date of birth of the individual - best estimate | in yyyy-mm-dd format | If actual month and day are not known it is OK to impute, e.g. assign to middle of the month or mid-year. |
| DoBEstimated | Indicates if date of birth of the individual provided is estimated | 0 No  1 Yes |  |
| DoD | The date of death of the individual, if the individual has died during the study period | in yyyy-mm-dd format |  |
| DoDEstimated | Indicates if date of death of the individual provided is estimated | 0 No  1 Yes |  |

***Table 2: Individuals Events***

| **Variable name** | **Description** | **Coding** | **Notes** |
| --- | --- | --- | --- |
| hdss_name | Name of HDSS site | site specific | Character |
| IndividualId | A number uniquely identifying all the records belonging to a specific individual in the data file. | site specific | Same as IndividualId in Individuals dataset |
| EventCode | A code identifying the type of event that has occurred. | (Refer to Table 3) |  |
| EventDate | The date on which the event occurred | in yyyy-mm-dd format | If actual month and day are not known it is OK to impute, e.g. assign to middle of the month or mid-year. |
| EventDateEstimated | Indicates if date on which the event occurred is estimated | 0 No  1 Yes |  |
| EventNr | Event number |  | A number increasing from 1 to EventCount for each event record in order of event occurrence |
| ObservationDate | Observation date |  | Date on which the event was observed (recorded), also known as surveillance visit date |
| LocationId | Location Identifier |  | Unique identifier associated with a residential unit within the site and is the location where the individual was or became resident when the event occurred. For data anonymisation purposes, this identifier should not be the same as the identifier used internally by the contributing centre, but the contributing centre should retain a mapping of this identifier to their internal location identifier. |

***Table 3: Events***

| **Code** | **Event** | **Definition** |
| --- | --- | --- |
| BTH | Birth | The birth of an individual to a resident female. |
| ENU | Enumeration | Starting event for all individuals present at the baseline census of the surveillance area. It is the date on which the individual was first observed to be present in the surveillance area during the baseline census. |
| IMG | In-migration | The event of migrating into the surveillance area |
| OMG | Out-migration | The event of migrating out of the surveillance area |
| EXT | Location exit | The event of leaving a residential location within the surveillance area to take up residence in another residential location within the surveillance area |
| ENT | Location entry | The event of taking up residence in a residential location within the surveillance area following a location exit event. Note that location exit and entry are actually two parts of the same action of changing residential location and as such happens on the same event date. |
| OBE | Observation end | An event inserted when a dataset is right censored at an arbitrary date and this individual remained under surveillance beyond this date. The right censor date is the date of this event |
| OBL | Last Observation | An event indicating the last point in time on which this individual was observed to be present and under surveillance. Event date equals observation date in this instance. Normally there should be no individuals with this event as their last event if the right censoring date is prior to the start of the last complete census round. |

1. **Cause of death**

- Mortality analyses by cause will be based on the Individual Event Histories and Verbal Autopsy data.
- Prior to attending the data preparation workshop, sites will need to extract from their most detailed and complete version of their verbal autopsy data into a common format that will be transformed into a format that allows to be processed with InterVA5, InsilicoVA and Tariff automated VA algorithms for assigning causes of death as specified in Table 4. The specification in the table is based on indicators which are required for assigning causes of death by the InterVA5 algorithm (<http://www.byass.uk/interva/>) but is also compatible to the indicators required for assigning causes of death by InsilicoVA (<https://github.com/verbal-autopsy-software/InSilicoVA>) and Tariff (<https://www.healthdata.org/verbal-autopsy/tools>) algorithms.

***Table 4: Verbal Autopsy data***

***Data for each death – one record per death, include children***

***Note: IndividualId should be the same as in residence episodes dataset***

| **Variable** | **Description** | **Corresponding question in WHO-2016 VA standard** | **Coding** | **Notes** |
| --- | --- | --- | --- | --- |
| hdss_name | Name of HDSS site |  |  |  |
| IndividualId | A number uniquely identifying all the records belonging to a specific individual in the data file. |  |  |  |
|  |  |  |  |  |
| interview_date | Date of VA interview |  |  |  |
| interviewer_id | ID number for the interviewer |  |  |  |
| dob | Date of birth- best estimate |  |  |  |
| dod | Date of death- best estimate |  |  |  |
| sex | Male or female |  | 1 Male  2 Female |  |
| i004a | Did s(he) die during the wet season? | id10004 | y yes  n no  leave blank “” if question not asked or answer not known |  |
| i004b | Did s(he) die during the dry season? | id10004 | -‘’- |  |
| i019a | Was he male? | id10019 | -‘’- |  |
| i019b | Was she female? | id10019 | -‘’- |  |
| i022a | Was s(he) aged 65 years or more at death? | id10022 | -‘’- |  |
| i022b | Was s(he) aged 50 to 64 years at death? | id10022 | -‘’- |  |
| i022c | Was s(he) aged 15 to 49 years at death? | id10022 | -‘’- |  |
| i022d | Was s(he) aged 5-14 years at death? | id10022 | -‘’- |  |
| i022e | Was s(he) aged 1 to 4 years at death? | id10022 | -‘’- |  |
| i022f | Was s(he) aged 1 to 11 months at death? | id10022 | -‘’- |  |
| i022g | Was s(he) aged < 1 month (28 days) at death? | id10022 | -‘’- |  |
| i022h | Was s(he) a live baby who died within 24 hours of birth? | id10022 | -‘’- |  |
| i022i | Was s(he) a baby who died between 24 and 48 hours of birth? | id10022 | -‘’- |  |
| i022j | Was s(he) a baby who died more than 48 hours from birth, but within the first week? | id10022 | -‘’- |  |
| i022k | Was s(he) a baby who died after the first week, but within the first month? | id10022 | -‘’- |  |
| i022l | Was she a woman aged 12-19 years at death? | id10022 | -‘’- |  |
| i022m | Was she a woman aged 20-34 years at death? | id10022 | -‘’- |  |
| i022n | Was she a woman aged 35 to 49 years at death? | id10022 | -‘’- |  |
| i059o | Was she married at the time of death? | id10059 | -‘’- |  |
| i077o | Did (s)he suffer from any injury or accident that led to her/his death? | id10077 | -‘’- |  |
| i079o | Was (s)he injured in a road traffic accident? | id10079 | -‘’- |  |
| i082o | Was (s)he injured in a non-road transport accident? | id10082 | -‘’- |  |
| i083o | Was (s)he injured in a fall? | id10083 | -‘’- |  |
| i084o | Was (s)he poisoned in any way? | id10084 | -‘’- |  |
| i085o | Did (s)he die of drowning? | id10085 | -‘’- |  |
| i086o | Was (s)he injured by the bite or sting of a venomous animal? | id10086 | -‘’- |  |
| i087o | Was (s)he injured by an animal or insect (non-venomous) | id10087 | -‘’- |  |
| i089o | Was (s)he injured by burns or fire? | id10089 | -‘’- |  |
| i090o | Was (s)he subject to violence (suicide, homicide,abuse)? | id10090 | -‘’- |  |
| i091o | Was (s)he injured by a fire arm? | id10091 | -‘’- |  |
| i092o | Was (s)he stabbed, cut or pierced? | id10092 | -‘’- |  |
| i093o | Was (s)he strangled? | id10093 | -‘’- |  |
| i094o | Was (s)he injured by a blunt force? | id10094 | -‘’- |  |
| i095o | Was (s)he injured by a force of nature? | id10095 | -‘’- |  |
| i096o | Was (s)he electrocuted? | id10096 | -‘’- |  |
| i098o | Was the injury accidental? | id10098 | -‘’- |  |
| i099o | Was the injury or accident self-inflicted? | id10099 | -‘’- |  |
| i100o | Was the injury or accident intentionally inflicted by someone else? | id10100 | -‘’- |  |
| i104o | Did the baby ever cry? | id10104 | -‘’- |  |
| i105o | Did the baby cry immediately after birth, even if only a little bit? | id10105 | -‘’- |  |
| i106a | Was it more than 5 minutes after birth before the baby first cried? | id10106 | -‘’- |  |
| i107o | Did the baby stop being able to cry? | id10107 | -‘’- |  |
| i108a | Did the baby stop crying more than a day before (s)he died? | id10108 | -‘’- |  |
| i109o | Did the baby ever move? | id10109 | -‘’- |  |
| i110o | Did the baby ever breathe? | id10110 | -‘’- |  |
| i111o | Did the baby breathe immediately after birth, even a little? | id10111 | -‘’- |  |
| i112o | Did the baby have a breathing problem? | id10112 | -‘’- |  |
| i113o | Was the baby given assistance to breathe at birth? | id10113 | -‘’- |  |
| i114o | If the baby didn't show any sign of life, was it born dead? | id10114 | -‘’- |  |
| i115o | Were there any bruises or signs of injury on child's body after the birth? | id10115 | -‘’- |  |
| i116o | Was the baby's body soft, pulpy and discoloured with the skin peeling away? | id10116 | -‘’- |  |
| i120a | Did the final illness last less than 3 weeks? | id10120 | -‘’- |  |
| i120b | Did the final illness last at least 3 weeks? | id10120 | -‘’- |  |
| i123o | Did (s)he die suddenly? | id10123 | -‘’- |  |
| i125o | Was there any diagnosis by a health professional of tuberculosis? | id10125 | -‘’- |  |
| i127o | Was there any diagnosis by a health professional of HIV/AIDS? | id10127 | -‘’- |  |
| i128o | Did (s)he have a recent positive test by a health professional for malaria? | id10128 | -‘’- |  |
| i129o | Did (s)he have a recent negative test by a health professional for malaria? | id10129 | -‘’- |  |
| i130o | Was there any diagnosis by a health professional of dengue fever? | id10130 | -‘’- |  |
| i131o | Was there any diagnosis by a health professional of measles? | id10131 | -‘’- |  |
| i132o | Was there any diagnosis by a health professional of high blood pressure? | id10132 | -‘’- |  |
| i133o | Was there any diagnosis by a health professional of heart disease? | id10133 | -‘’- |  |
| i134o | Was there any diagnosis by a health professional of diabetes? | id10134 | -‘’- |  |
| i135o | Was there any diagnosis by a health professional of asthma? | id10135 | -‘’- |  |
| i136o | Was there any diagnosis by a health professional of epilepsy? | id10136 | -‘’- |  |
| i137o | Was there any diagnosis by a health professional of cancer? | id10137 | -‘’- |  |
| i138o | Was there any diagnosis by a health professional of Chronic Obstructive Pulmonary Disease (COPD)? | id10138 | -‘’- |  |
| i139o | Was there any diagnosis by a physician or health worker of dementia? | id10139 | -‘’- |  |
| i140o | Was there any diagnosis by a health professional of depression? | id10140 | -‘’- |  |
| i141o | Was there any diagnosis by a health professional of stroke? | id10141 | -‘’- |  |
| i142o | Was there any diagnosis by a health professional of sickle cell disease? | id10142 | -‘’- |  |
| i143o | Was there any diagnosis by a health professional of kidney disease? | id10143 | -‘’- |  |
| i144o | Was there any diagnosis by a health professional of liver disease? | id10144 | -‘’- |  |
| i147o | During the illness that led to death, did (s)he have a fever? | id10147 | -‘’- |  |
| i148a | Did the fever last less than a week before death? | id10148 | -‘’- |  |
| i148b | Did the fever last at least one week, but less than 2 weeks before death? | id10148 | -‘’- |  |
| i148c | Did the fever last at least 2 weeks before death? | id10148 | -‘’- |  |
| i149o | Did the fever continue until death? | id10149 | -‘’- |  |
| i150a | Was the fever severe? | id10150 | -‘’- |  |
| i151a | Was the fever continuous? | id10151 | -‘’- |  |
| i152o | Did (s)he have night sweats? | id10152 | -‘’- |  |
| i153o | During the illness that led to death, did (s)he have a cough? | id10153 | -‘’- |  |
| i154a | Did the cough last less than 3 weeks before death? | id10154 | -‘’- |  |
| i154b | Did the cough last at least 3 weeks before death? | id10154 | -‘’- |  |
| i155o | Was the cough productive, with sputum? | id10155 | -‘’- |  |
| i156o | Was the cough very severe? | id10156 | -‘’- |  |
| i157o | Did (s)he cough up blood? | id10157 | -‘’- |  |
| i158o | Did (s)he make a whooping sound when coughing? | id10158 | -‘’- |  |
| i159o | During the illness that led to death, did (s)he have any difficulty breathing? | id10159 | -‘’- |  |
| i161a | Did the difficult breathing last for at least 3 days before death? | id10161 | -‘’- |  |
| i165a | Was the difficult breathing continuous during this period? | id10165 | -‘’- |  |
| i166o | Did (s)he have fast breathing? | id10166 | -‘’- |  |
| i167a | Did the fast breathing last for less than two weeks before death? | id10167 | -‘’- |  |
| i167b | Did the fast breathing last for at least 2 weeks before death? | id10167 | -‘’- |  |
| i168o | Did (s)he have breathlessness? | id10168 | -‘’- |  |
| i169a | Did the breathlessness last for less than 2 weeks before death? | id10169 | -‘’- |  |
| i169b | Did the breathlessness last for at least 2 weeks before death? | id10169 | -‘’- |  |
| i170o | Was (s)he unable to carry out daily routines due to breathlessness? | id10170 | -‘’- |  |
| i171o | Was (s)he breathless while lying flat? | id10171 | -‘’- |  |
| i172o | Did you see the lower chest wall/ribs being pulled in as the child breathed? | id10172 | -‘’- |  |
| i173a | Did his/her breathing sound like wheezing or grunting? | id10173 | -‘’- |  |
| i174o | During the illness that led to death, did (s)he have chest pain? | id10174 | -‘’- |  |
| i175o | Was the chest pain severe? | id10175 | -‘’- |  |
| i176a | Did (s)he experience chest pain at least 3 days before death? | id10176 | -‘’- |  |
| i178a | Did the chest pain last for at least 30 minutes? | id10178 | -‘’- |  |
| i181o | Did (s)he have diarrhoea? | id10181 | -‘’- |  |
| i182a | Did (s)he have diarrhoea for less than 2 weeks before death? | id10182 | -‘’- |  |
| i182b | Did (s)he have diarrhoea for at least 2 weeks but less than 4 weeks before death? | id10182 | -‘’- |  |
| i182c | Did (s)he have diarrhoea for at least 4 weeks before death? | id10182 | -‘’- |  |
| i183a | Did the baby or child have at least 4 stools on the day that loose liquid stools were most frequent? | id10183 | -‘’- |  |
| i184a | Did the frequent loose or liquid stools start at least 3 days before death? | id10184 | -‘’- |  |
| i185o | Did the frequent loose or liquid stools continue up until death? | id10185 | -‘’- |  |
| i186o | At any time during the final illness was there blood in the stools? | id10186 | -‘’- |  |
| i187o | Was there blood in the stool up until death? | id10187 | -‘’- |  |
| i188o | During the illness that led to death, did (s)he vomit? | id10188 | -‘’- |  |
| i189o | Did (s)he vomit in the week preceding the death? | id10189 | -‘’- |  |
| i190o | Did (s)he vomit for at least 3 days before death? | id10190 | -‘’- |  |
| i191o | Was there blood in the vomit? | id10191 | -‘’- |  |
| i192o | Was the vomit black? | id10192 | -‘’- |  |
| i193o | During the illness that led to death, did (s)he have any abdominal (belly) problem? | id10193 | -‘’- |  |
| i194o | Did (s)he have abdominal pain? | id10194 | -‘’- |  |
| i195o | Was the abdominal pain severe? | id10195 | -‘’- |  |
| i197a | Did (s)he have severe abdominal pain for less than 2 weeks before death? | id10197 | -‘’- |  |
| i197b | Did (s)he have severe abdominal pain for at least 2 weeks before death? | id10197 | -‘’- |  |
| i199a | Was the pain in the upper abdomen? | id10199 | -‘’- |  |
| i199b | Was the pain in the lower abdomen? | id10199 | -‘’- |  |
| i200o | Did (s)he have a more than usually protruding abdomen? | id10200 | -‘’- |  |
| i201a | Did (s)he have a more than usually protruding abdomen for less than 2 weeks before death? | id10201 | -‘’- |  |
| i201b | Did (s)he have a more than usually protruding abdomen for at least 2 weeks before death? | id10201 | -‘’- |  |
| i203a | Did (s)he develop the protruding abdomen rapidly? | id10203 | -‘’- |  |
| i204o | Did (s)he have any mass in the abdomen? | id10204 | -‘’- |  |
| i205a | Did (s)he have a mass in the abdomen for less than 2 weeks before death? | id10205 | -‘’- |  |
| i205b | Did (s)he have a mass in the abdomen for at least 2 weeks before death? | id10205 | -‘’- |  |
| i207o | During the illness that led to death, did (s)he have a severe headache? | id10207 | -‘’- |  |
| i208o | During the illness that led to death, did (s)he have a stiff neck? | id10208 | -‘’- |  |
| i209a | Did (s)he have a stiff neck for less than one week before death? | id10209 | -‘’- |  |
| i209b | Did (s)he have a stiff neck for at least one week before death? | id10209 | -‘’- |  |
| i210o | During the illness that led to death, did (s)he have a painful neck? | id10210 | -‘’- |  |
| i211a | Did (s)he have a painful neck for at least one week before death? | id10211 | -‘’- |  |
| i212o | During the illness that led to death, did (s)he have mental confusion? | id10212 | -‘’- |  |
| i213o | Did (s)he have mental confusion for at least 3 months before death? | id10213 | -‘’- |  |
| i214o | During the illness that led to death, was (s)he unconscious? | id10214 | -‘’- |  |
| i215o | Was (s)he unconscious for at least 24 hours before death? | id10215 | -‘’- |  |
| i216a | Was (s)he unsconscious for at least 6 hours before death? | id10216 | -‘’- |  |
| i217o | Did the unconsciousness start suddenly, quickly (at least within a single day)? | id10217 | -‘’- |  |
| i218o | Did the unconsciousness continue until death? | id10218 | -‘’- |  |
| i219o | During the illness that led to death, did (s)he have any convulsions? | id10219 | -‘’- |  |
| i220o | Did (s)he experience any generalized convulsions or fits? | id10220 | -‘’- |  |
| i221a | Did the convulsions last for less than 10 minutes? | id10221 | -‘’- |  |
| i221b | Did the convulsions last for at least 10 minutes? | id10221 | -‘’- |  |
| i222o | Did (s)he become unconscious immediately after the convulsion? | id10222 | -‘’- |  |
| i223o | During the illness that led to death, did (s)he have any urine problems? | id10223 | -‘’- |  |
| i224o | Did (s)he stop urinating? | id10224 | -‘’- |  |
| i225o | Did (s)he go to urinate more often than usual? | id10225 | -‘’- |  |
| i226o | Did (s)he pass blood in the urine? | id10226 | -‘’- |  |
| i227o | During the illness that led to death, did (s)he have any sores or ulcers anywhere? | id10227 | -‘’- |  |
| i228o | Did (s)he have sores? | id10228 | -‘’- |  |
| i229o | Did the sores have clear fluid and/or pus? | id10229 | -‘’- |  |
| i230o | Did (s)he have an ulcer (pit) on the foot? | id10230 | -‘’- |  |
| i231o | Did any ulcer ooze pus? | id10231 | -‘’- |  |
| i232a | Did the ulcer ooze pus for at least 2 weeks? | id10232 | -‘’- |  |
| i233o | During the illness that led to death, did (s)he have any skin rash? | id10233 | -‘’- |  |
| i234a | Did (s)he have the skin rash for less than one week? | id10234 | -‘’- |  |
| i234b | Did (s)he have the skin rash for at least one week? | id10234 | -‘’- |  |
| i235a | Did (s)he have a rash on the face? | id10235 | -‘’- |  |
| i235b | Did (s)he have a rash on the trunk or abdomen? | id10235 | -‘’- |  |
| i235c | Did (s)he have a rash on the extremities? | id10235 | -‘’- |  |
| i235d | Did (s)he have a rash everywhere? | id10235 | -‘’- |  |
| i236o | Did (s)he have measles rash? | id10236 | -‘’- |  |
| i237o | Did (s)he ever have shingles or herpes zoster? | id10237 | -‘’- |  |
| i238o | During the illness that led to death, did her/his skin flake off in patches? | id10238 | -‘’- |  |
| i239o | During the illness that led to death, did he/she have areas of the skin that turned black? | id10239 | -‘’- |  |
| i240o | During the illness that led to death, did he/she have areas of the skin with redness and swelling? | id10240 | -‘’- |  |
| i241o | During the illness that led to death, did (s)he bleed from anywhere? | id10241 | -‘’- |  |
| i242o | Did (s)he bleed from the nose, mouth or anus? | id10242 | -‘’- |  |
| i243o | During the illness that led to death, did (s)he have noticeable weight loss? | id10243 | -‘’- |  |
| i244o | Was (s)he severely thin or wasted? | id10244 | -‘’- |  |
| i245o | During the illness that led to death, did s/he have a whitish rash inside the mouth or on the tongue? | id10245 | -‘’- |  |
| i246o | During the illness that led to death, did (s)he have stiffness of the whole body or was unable to open the mouth? | id10246 | -‘’- |  |
| i247o | During the illness that led to death, did (s)he have puffiness of the face? | id10247 | -‘’- |  |
| i248a | Did (s)he have puffiness of the face for at least one week before death? | id10248 | -‘’- |  |
| i249o | During the illness that led to death, did (s)he have swollen legs or feet? | id10249 | -‘’- |  |
| i250a | Did the swelling last for at least 3 days before death? | id10250 | -‘’- |  |
| i251o | Did (s)he have both feet swollen? | id10251 | -‘’- |  |
| i252o | During the illness that led to death, did (s)he have general puffiness all over his/her body? | id10252 | -‘’- |  |
| i253o | During the illness that led to death, did (s)he have any lumps? | id10253 | -‘’- |  |
| i254o | Did (s)he have any lumps or lesions in the mouth? | id10254 | -‘’- |  |
| i255o | Did (s)he have any lumps in the neck? | id10255 | -‘’- |  |
| i256o | Did (s)he have any lumps in the armpit? | id10256 | -‘’- |  |
| i257o | Did (s)he have any lumps in the groin? | id10257 | -‘’- |  |
| i258o | During the illness that led to death, was (s)he in any way paralysed? | id10258 | -‘’- |  |
| i259o | Did (s)he have paralysis of only one side of the body? | id10259 | -‘’- |  |
| i260a | Was only the right side of the body paralysed? | id10260 | -‘’- |  |
| i260b | Was only the left side of the body paralysed? | id10260 | -‘’- |  |
| i260c | Was only the lower part of the body paralysed? | id10260 | -‘’- |  |
| i260d | Was only the upper part of the body paralysed? | id10260 | -‘’- |  |
| i260e | Was only one leg paralysed? | id10260 | -‘’- |  |
| i260f | Was only one arm paralysed? | id10260 | -‘’- |  |
| i260g | Was the entire body paralysed? | id10260 | -‘’- |  |
| i261o | During the illness that led to death, did (s)he have difficulty swallowing? | id10261 | -‘’- |  |
| i262a | Did (s)he have difficulty swallowing for at least one week before death? | id10262 | -‘’- |  |
| i263a | Did (s)he have difficulty with swallowing solids? | id10263 | -‘’- |  |
| i263b | Did (s)he have difficulty with swallowing liquids? | id10263 | -‘’- |  |
| i264o | Did (s)he have pain upon swallowing? | id10264 | -‘’- |  |
| i265o | During the illness that led to death, did (s)he have yellow discolouration of the eyes? | id10265 | -‘’- |  |
| i266a | Did (s)he have the yellow discolouration for at least 3 weeks before death? | id10266 | -‘’- |  |
| i267o | During the illness that led to death, did her/his hair change to a reddish or yellowish colour? | id10267 | -‘’- |  |
| i268o | During the illness that led to death, did (s)he look pale (thinning/lack of blood) or have pale palms, eyes or nail beds? | id10268 | -‘’- |  |
| i269o | During the illness that led to death, did (s)he have sunken eyes? | id10269 | -‘’- |  |
| i270o | During the illness that led to death, did (s)he drink a lot more water than usual? | id10270 | -‘’- |  |
| i271o | Was the baby able to suckle or bottle-feed within the first 24 hours after birth? | id10271 | -‘’- |  |
| i272o | Did the baby ever suckle in a normal way? | id10272 | -‘’- |  |
| i273o | Did the baby stop suckling? | id10273 | -‘’- |  |
| i274a | Did the baby stop suckling on the 2nd day of life or later? | id10274 | -‘’- |  |
| i275o | Did the baby have convulsions starting within the first 24 hours of life? | id10275 | -‘’- |  |
| i276o | Did the baby have convulsions starting more than 24 hours after birth? | id10276 | -‘’- |  |
| i277o | Did the baby's body become stiff, with the back arched backwards? | id10277 | -‘’- |  |
| i278o | During the illness that led to death, did the baby have a bulging or raised fontanelle? | id10278 | -‘’- |  |
| i279o | During the illness that led to death, did the baby have a sunken fontanelle? | id10279 | -‘’- |  |
| i281o | During the illness that led to death, did the baby become unresponsive or unconscious? | id10281 | -‘’- |  |
| i282o | Did the baby become unresponsive or unconscious soon after birth, within less than 24 hours? | id10282 | -‘’- |  |
| i283o | Did the baby become unresponsive or unconscious more than 24 hours after birth? | id10283 | -‘’- |  |
| i284o | During the illness that led to death, did the baby become cold to touch? | id10284 | -‘’- |  |
| i285a | Was the baby more than 3 days old when it started feeling cold to touch? | id10285 | -‘’- |  |
| i286o | During the illness that led to death, did the baby become lethargic, after a period of normal activity? | id10286 | -‘’- |  |
| i287o | Did the baby have redness or discharge from the umbilical cord stump? | id10287 | -‘’- |  |
| i288o | During the illness that led to death, did the baby have skin ulcer(s) or pits? | id10288 | -‘’- |  |
| i289o | During the illness that led to death, did the baby have yellow skin, palms (hand) or soles (foot)? | id10289 | -‘’- |  |
| i290o | Did the baby or infant appear to be healthy and then just die suddenly? | id10290 | -‘’- |  |
| i294o | During the illness that led to death, did she have any swelling or lump in the breast? | id10294 | -‘’- |  |
| i295o | During the illness that led to death, did she have any ulcers (pits) in the breast? | id10295 | -‘’- |  |
| i296o | Did she ever have a period or menstruate? | id10296 | -‘’- |  |
| i297o | During the illness that led to death, did she have excessive vaginal bleeding in between menstrual periods? | id10297 | -‘’- |  |
| i298o | Was the bleeding excessive? | id10298 | -‘’- |  |
| i299o | Did her menstrual period stop naturally because of menopause? | id10299 | -‘’- |  |
| i300o | Did she have vaginal bleeding after cessation of menstruation? | id10300 | -‘’- |  |
| i301o | Was there excessive vaginal bleeding in the week prior to death? | id10301 | -‘’- |  |
| i302o | At the time of death was her period overdue? | id10302 | -‘’- |  |
| i303a | Had her period been overdue for at least 4 weeks? | id10303 | -‘’- |  |
| i304o | Did she have a sharp pain in her abdomen shortly before death? | id10304 | -‘’- |  |
| i305o | Was she pregnant at the time of death? | id10305 | -‘’- |  |
| i306o | Did she die within 6 weeks of delivery, abortion or miscarriage? | id10306 | -‘’- |  |
| i309o | Was she, or had she been, pregnant for less than 6 months when she died? | id10309 | -‘’- |  |
| i310o | Please confirm: When she died, she was NEITHER pregnant NOR had recently been pregnant NOR had recently delivered when she died - is that right? | id10310 | -‘’- |  |
| i312o | Did she die during labour, but before delivery? | id10312 | -‘’- |  |
| i313o | Did she die after delivering a baby? | id10313 | -‘’- |  |
| i314o | Did she die within 24 hours after delivery? | id10314 | -‘’- |  |
| i315o | Did she die within 6 weeks of childbirth? | id10315 | -‘’- |  |
| i316o | Did she give birth to a live baby (within 6 weeks of her death)? | id10316 | -‘’- |  |
| i317o | Did she die during or after a multiple pregnancy? | id10317 | -‘’- |  |
| i318o | Was she breastfeeding the child in the days before death? | id10318 | -‘’- |  |
| i319a | Did she die during or after her first pregnancy? | id10319 | -‘’- |  |
| i319b | Did she have four or more pregnancies before this one? | id10319 | -‘’- |  |
| i320o | Had she had any previous Caesarean section? | id10320 | -‘’- |  |
| i321o | During pregnancy, did she suffer from high blood pressure? | id10321 | -‘’- |  |
| i322o | Did she have foul smelling vaginal discharge during pregnancy or after delivery? | id10322 | -‘’- |  |
| i323o | During the last 3 months of pregnancy, did she suffer from convulsions? | id10323 | -‘’- |  |
| i324o | During the last 3 months of pregnancy did she suffer from blurred vision? | id10324 | -‘’- |  |
| i325o | Did she have excessive bleeding during pregnancy or shortly after delivery? | id10325 | -‘’- |  |
| i326o | Was there vaginal bleeding during the first 6 months of pregnancy? | id10326 | -‘’- |  |
| i327o | Was there vaginal bleeding during the last 3 months of pregnancy but before labour started? | id10327 | -‘’- |  |
| i328o | Did she have excessive bleeding during labour, before delivery? | id10328 | -‘’- |  |
| i329o | Did she have excessive bleeding after delivery or abortion? | id10329 | -‘’- |  |
| i330o | Was the placenta completely delivered? | id10330 | -‘’- |  |
| i331o | Did she deliver or try to deliver an abnormally positioned baby? | id10331 | -‘’- |  |
| i332a | Did her labour last longer than 24 hours? | id10332 | -‘’- |  |
| i333o | Did she attempt to terminate the pregnancy? | id10333 | -‘’- |  |
| i334o | Did she recently have a pregnancy that ended in an abortion (spontaneous or induced)? | id10334 | -‘’- |  |
| i335o | Did she die during an abortion? | id10335 | -‘’- |  |
| i336o | Did she die within 6 weeks of having an abortion? | id10336 | -‘’- |  |
| i337a | Did the mother deliver at a health facility or clinic? | id10337 | -‘’- |  |
| i337b | Did the mother deliver at home? | id10337 | -‘’- |  |
| i337c | Did the mother deliver elsewhere (not at a health facility nor at home)? | id10337 | -‘’- |  |
| i338o | Did she receive professional assistance during the delivery? | id10338 | -‘’- |  |
| i340o | Did she have an operation to remove her uterus shortly before death? | id10340 | -‘’- |  |
| i342o | Was the delivery normal vaginal, without forceps or vacuum? | id10342 | -‘’- |  |
| i343o | Was the delivery vaginal, with forceps or vacuum? | id10343 | -‘’- |  |
| i344o | Was the delivery a Caesarean section? | id10344 | -‘’- |  |
| i347o | Was her baby born more than one month early? | id10347 | -‘’- |  |
| i354o | Was the child part of a multiple birth? | id10354 | -‘’- |  |
| i355a | If the child was part of a multiple birth, was it born first? | id10355 | -‘’- |  |
| i356o | Is the child's mother still alive? | id10356 | -‘’- |  |
| i357o | Did the child's mother die during or shortly after the delivery? | id10357 | -‘’- |  |
| i358a | Did the child's mother die in the baby's first year of life? | id10358 | -‘’- |  |
| i360a | Was the baby born in a health facility or clinic? | id10360 | -‘’- |  |
| i360b | Was the baby born at home? | id10360 | -‘’- |  |
| i360c | Was the baby born somewhere else (e.g. on the way to a clinic)? | id10360 | -‘’- |  |
| i361o | Did the mother receive professional assistance during the delivery? | id10361 | -‘’- |  |
| i362o | At birth, was the baby of usual size? | id10362 | -‘’- |  |
| i363o | At birth, was the baby smaller than normal (weighing under 2.5 kg)? | id10363 | -‘’- |  |
| i364o | At birth, was the baby very much smaller than usual, (weighing under 1 kg)? | id10364 | -‘’- |  |
| i365o | At birth, was the baby larger than normal (weighing over 4.5 kg)? | id10365 | -‘’- |  |
| i367a | Was the baby born during the ninth month (at least 37 weeks) of pregnancy? | id10367 | -‘’- |  |
| i367b | Was the baby born during the eighth month (34 to 37 weeks) of pregnancy? | id10367 | -‘’- |  |
| i367c | Was the baby born before the eighth month (less than 34 weeks) of pregnancy? | id10367 | -‘’- |  |
| i368o | Were there any complications in the late part of the pregnancy (defined as the last 3 months), but before labour? | id10368 | -‘’- |  |
| i369o | Were there any complications during labour or delivery? | id10369 | -‘’- |  |
| i370o | Was any part of the baby physically abnormal at time of delivery? (for example: body part too large or too small, additional growth on body)? | id10370 | -‘’- |  |
| i371o | Did the baby/child have a swelling or defect on the back? | id10371 | -‘’- |  |
| i372o | Did the baby/child have a very large head? | id10372 | -‘’- |  |
| i373o | Did the baby/child have a very small head? | id10373 | -‘’- |  |
| i376o | Was the baby moving in the last few days before the birth? | id10376 | -‘’- |  |
| i377o | Did the baby stop moving in the womb before labour started? | id10377 | -‘’- |  |
| i382a | Did labour and delivery take more than 24 hours? | id10382 | -‘’- |  |
| i383o | Was the baby born 24 hours or more after the waters broke? | id10383 | -‘’- |  |
| i384o | Was the liquor foul smelling when the waters broke? | id10384 | -‘’- |  |
| i385a | Was the liquor a green or brown colour when the waters broke? | id10385 | -‘’- |  |
| i387o | Was the delivery normal vaginal, without forceps or vacuum? | id10387 | -‘’- |  |
| i388o | Was the delivery vaginal, with forceps or vacuum? | id10388 | -‘’- |  |
| i389o | Was the delivery a Caesarean section? | id10389 | -‘’- |  |
| i391o | Did the child's mother receive any vaccinations since reaching adulthood including during this pregnancy? | id10391 | -‘’- |  |
| i393o | Did the mother receive tetanus toxoid (TT) vaccine? | id10393 | -‘’- |  |
| i394a | Was this baby born from the mother's first pregnancy? | id10394 | -‘’- |  |
| i394b | Did the baby's mother have four or more births before this one? | id10394 | -‘’- |  |
| i395o | During labour, did the baby's mother suffer from fever? | id10395 | -‘’- |  |
| i396o | During the last 3 months of pregnancy, labour or delivery, did the baby's mother suffer from high blood pressure? | id10396 | -‘’- |  |
| i397o | Did the baby's mother have diabetes mellitus? | id10397 | -‘’- |  |
| i398o | Did the baby's mother have foul smelling vaginal discharge during pregnancy or after delivery? | id10398 | -‘’- |  |
| i399o | During the last 3 months of pregnancy, labour or delivery, did the baby's mother suffer from convulsions? | id10399 | -‘’- |  |
| i400o | During the last 3 months of pregnancy did the baby's mother suffer from blurred vision? | id10400 | -‘’- |  |
| i401o | Did the baby's mother have severe anaemia? | id10401 | -‘’- |  |
| i402o | Did the baby's mother have vaginal bleeding during the last 3 months of pregnancy but before labour started? | id10402 | -‘’- |  |
| i403o | Did the baby's bottom, feet, arm or hand come out of the vagina before its head? | id10403 | -‘’- |  |
| i404o | Was the umbilical cord wrapped more than once around the baby's neck at birth? | id10404 | -‘’- |  |
| i405o | Was the umbilical cord delivered first? | id10405 | -‘’- |  |
| i406o | Was the baby blue in colour at birth? | id10406 | -‘’- |  |
| i408o | Before the illness that led to death, was the baby/child growing normally? | id10408 | -‘’- |  |
| i411o | Did (s)he drink alcohol? | id10411 | -‘’- |  |
| i412o | Did (s)he use tobacco? | id10412 | -‘’- |  |
| i413o | Did (s)he smoke tobacco (cigarette, cigar, pipe, etc.)? | id10413 | -‘’- |  |
| i414a | Did (s)he use non-smoking tobacco? | id10414 | -‘’- |  |
| i415a | Did (s)he smoke at least 10 cigarettes daily? | id10415 | -‘’- |  |
| i418o | Did (s)he receive any treatment for the illness that led to death? | id10418 | -‘’- |  |
| i419o | Did (s)he receive oral rehydration salts? | id10419 | -‘’- |  |
| i420o | Did (s)he receive (or need) intravenous fluids (drip) treatment? | id10420 | -‘’- |  |
| i421o | Did (s)he receive (or need) a blood transfusion? | id10421 | -‘’- |  |
| i422o | Did (s)he receive (or need) treatment/food through a tube passed through the nose? | id10422 | -‘’- |  |
| i423o | Did (s)he receive (or need) injectable antibiotics? | id10423 | -‘’- |  |
| i424o | Did (s)he receive (or need) antiretroviral therapy (ART)? | id10424 | -‘’- |  |
| i425o | Did (s)he have (or need) an operation for the illness? | id10425 | -‘’- |  |
| i426o | Did (s)he have the operation within 1 month before death? | id10426 | -‘’- |  |
| i427o | Was (s)he discharged from hospital very ill? | id10427 | -‘’- |  |
| i428o | Did (s)he receive appropriate immunizations? | id10428 | -‘’- |  |
| i450o | In the final days before death, did s/he travel to a hospital or health facility? | id10450 | -‘’- |  |
| i451o | Did (s)he use motorised transport to get to the hospital or health facility? | id10451 | -‘’- |  |
| i452o | Were there any problems during admission to the hospital or health facility? | id10452 | -‘’- |  |
| i453o | Were there any problems with the way (s)he was treated (medical treatment, procedures, interpersonal attitudes, respect, dignity) in the hospital or health facility? | id10453 | -‘’- |  |
| i454o | Were there any problems getting medications, or diagnostic tests in the hospital or health facility? | id10454 | -‘’- |  |
| i455o | Does it take more than 2 hours to get to the nearest hospital or health facility from the deceased's household? | id10455 | -‘’- |  |
| i456o | In the final days before death, were there any doubts about whether medical care was needed? | id10456 | -‘’- |  |
| i457o | In the final days before death, was traditional medicine used? | id10457 | -‘’- |  |
| i458o | In the final days before death, did anyone use a telephone or cell phone to call for help? | id10458 | -‘’- |  |
| i459o | Over the course of illness, did the total costs of care and treatment prohibit other household payments? | id10459 | -‘’- |  |

The site will also required to provide information for the identification of possible COVID-19 deaths based on the questions in the table below which were added to the WHO-2016 VA standard in 2020.

|  |  |
| --- | --- |
| **Description** | **Question in WHO-2016 VA standard** |
| Was there any diagnosis by a health professional of COVID-19? | Id10482 |
| Did s(h)e have a recent test by a health professional for COVID-19? | Id10483 |
| What was the result? | Id10484 |
| Did s(h)e suffer from extreme fatigue? | Id10485 |
| Did (s)he experience a new loss, change or decreased sense of smell or taste? | Id10486 |
| In the two weeks before death, did (s)he live with, visit, or care for someone who had any COVID-19 symptoms or a positive COVID-19 test? | Id10487 |
| In the two weeks before death, did (s)he travel to an area where COVID-19 is known to be present? | Id10488 |

1. **Data sharing**

Anonymised pooled data from the project will be deposited in an open data repository accessible to the wider research community.
